# Rheumatoid Pleuropulmonary Disease Mimicking Refractory Empyema: A Diagnostic Challenge With Trapped Lung

**DOI:** 10.7759/cureus.114568

**Published:** 2026-08-15

**Authors:** Diya Asad, Nagham Joudeh, Mohammad Jundy, Mohamed Ibraheem, Jehad Azar

**Affiliations:** 1 Medicine, Al-Quds University, Jerusalem, PSE; 2 Medicine, Faculty of Medicine, An-Najah National University, Nablus, PSE; 3 Medicine, Faculty of Medicine, University of Jordan, Amman, JOR; 4 Critical Care, Mayo Clinic, Phoenix, USA; 5 Transplantation, Mayo Clinic, Phoenix, USA; 6 Pulmonary and Critical Care, Mayo Clinic, Phoenix, USA

**Keywords:** case report, decortication, empyema, follicular bronchiolitis, interstitial lung disease, pleural effusion, rheumatoid arthritis, rheumatoid pleuritis, rituximab, usual interstitial pneumonia

## Abstract

Rheumatoid arthritis (RA)-related pleural disease is an uncommon but serious extra-articular complication that can closely mimic empyema. Rheumatoid pleural effusions are exudative, with low pH, markedly reduced glucose, and elevated lactate dehydrogenase (LDH), and the fluid may appear green and frankly purulent, a constellation that overlaps substantially with infected parapneumonic effusion, particularly when cultures are negative.

We report a 66-year-old woman with a 22-year history of seropositive, erosive, nodular RA who presented with recurrent right-sided pleural effusions and progressive dyspnea over three years. She was initially diagnosed with empyema following a presumed ruptured pleural-based pulmonary abscess. Pleural fluid was an exudate with elevated LDH (1,364 U/L), low glucose (45 mg/dL), and a lipid profile consistent with a chronic pseudochylous effusion, and repeated bacterial, mycobacterial, and fungal cultures were sterile. Video-assisted thoracoscopic surgery (VATS) with pleural and lung biopsies demonstrated findings characteristic of RA-related lung disease, including fibrinous pleuritis, follicular bronchiolitis, and a usual interstitial pneumonia (UIP) pattern. Because the lung remained trapped by a fibrous peel, staged thoracotomy with decortication and parietal pleurectomy was performed, and immunosuppression was escalated from leflunomide to rituximab.

Detailed long-term follow-up data were not available for inclusion in this report. This case illustrates that in a patient with established RA, an exudative, culture-negative, purulent-appearing pleural effusion should raise suspicion of rheumatoid pleuritis before empyema. Early recognition, alongside continued exclusion of infection, may reduce diagnostic delay and the need for extensive thoracic surgery.

## Introduction

Rheumatoid arthritis (RA) is a progressive systemic autoimmune disorder characterized by articular and extra-articular manifestations. The lung is a frequent site of extra-articular involvement, with disease affecting the airways, parenchyma, vasculature, and pleura [1,2]. Pleural involvement is among the most common thoracic manifestations of RA. Small pleural effusions have been identified in up to 70% of autopsy series, whereas clinically evident, symptomatic effusions occur in approximately 3%-5% of patients [3,4]. Proposed mechanisms include chronic pleural inflammation, local immune complex deposition, infection associated with immunosuppressive therapy, and drug-related pleural disease [3].

Rheumatoid pleural effusions can closely mimic empyema. They are typically exudative and may have a low pH, markedly reduced glucose concentration, and elevated lactate dehydrogenase (LDH). The fluid may also appear green or frankly purulent, even in the absence of infection [3,5]. When bacterial cultures are negative, the distinction from culture-negative pleural infection becomes particularly difficult [6]. We describe a patient with RA-related pleural and pulmonary disease who was initially diagnosed with empyema secondary to a presumed pleural-based pulmonary abscess. Video-assisted thoracoscopic biopsy ultimately demonstrated rheumatoid involvement of the pleura, small airways, and lung parenchyma.

This case was presented in abstract form at the American Thoracic Society International Conference, 2025.

## Case presentation

A 66-year-old woman presented with a three-year history of recurrent right-sided pleural effusion and progressive dyspnea. Her medical history was notable for seropositive, erosive, nodular RA diagnosed in 2002, with arthritis of the small joints of the hands and feet, peripheral neuropathy, and subcutaneous elbow nodules. She received methotrexate from 2002 to 2013 and was subsequently switched to leflunomide 20 mg daily.

**Figure 1:**
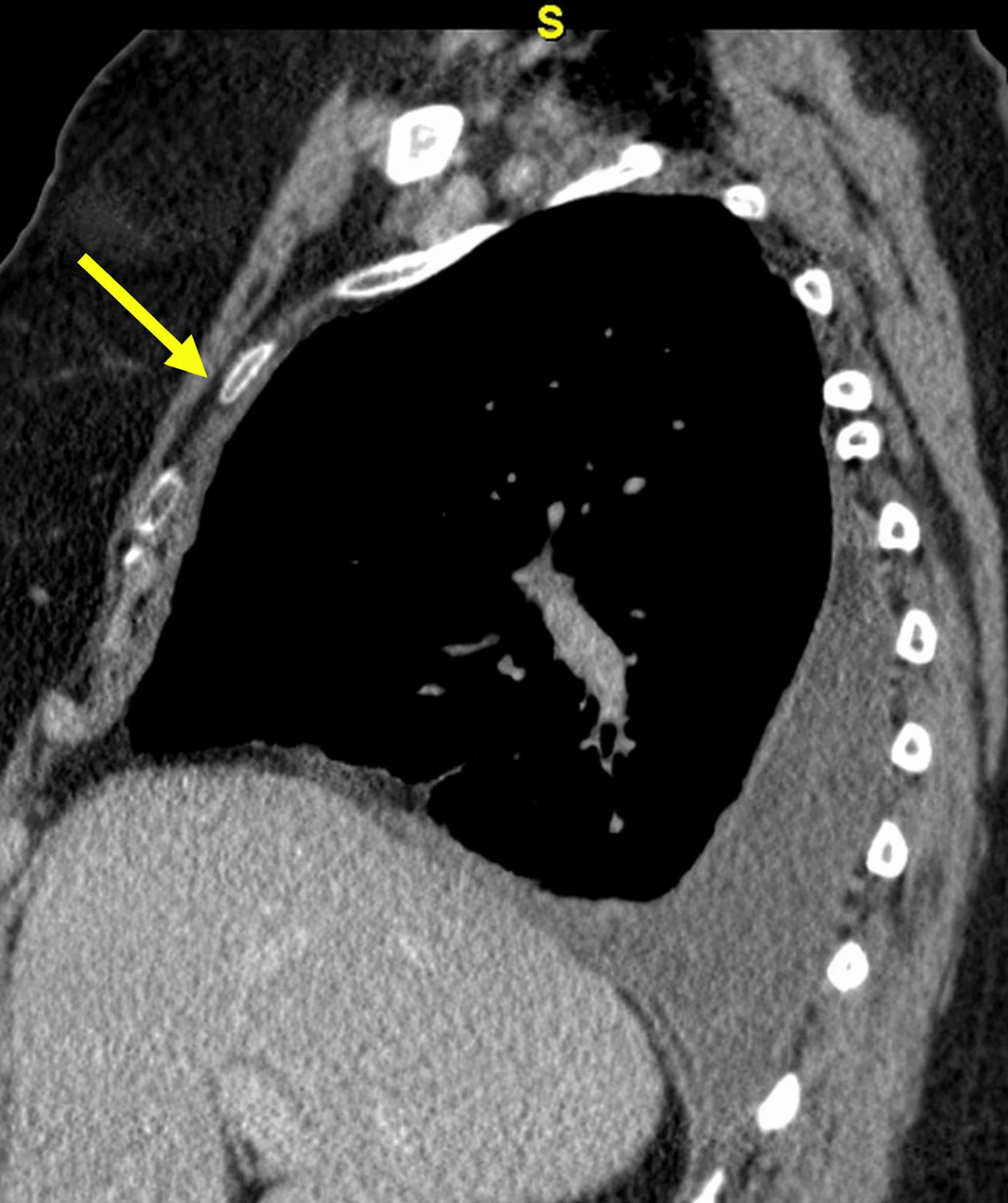
Sagittal CT demonstrating right pleural thickening

Her initial presentation in 2022 involved a large right-sided pleural effusion attributed to a ruptured pleural-based pulmonary abscess, leading to a diagnosis of empyema with a subsequent trapped lung (Figure 1). Thoracentesis yielded turbid, green, purulent-appearing fluid. Pleural fluid analysis demonstrated an exudate with elevated LDH (1,364 U/L) and low glucose (45 mg/dL; 2.5 mmol/L); the lipid profile showed cholesterol of 121 mg/dL exceeding triglycerides of 79 mg/dL, giving a cholesterol-to-triglyceride ratio above 1 and a triglyceride concentration below the 110 mg/dL threshold for chylothorax, a pattern suggestive of a chronic pseudochylous effusion (Table 1). Despite findings suggestive of an infectious process, bacterial, mycobacterial, and fungal cultures were all negative, and the effusion proved refractory to drainage.

**Table 1:** Pleural fluid analysis at initial presentation Light's criteria are used to distinguish exudative from transudative pleural effusions [7]. Pleural fluid pH, total protein, and nucleated cell count with differential were not available for review. U/L: units per liter; mg/dL: milligrams per deciliter; mmol/L: millimoles per liter

Parameter	Patient value	Reference/interpretive value	Interpretation
Appearance	Turbid, green, purulent-appearing	Clear, pale yellow	Abnormal
Lactate dehydrogenase	1,364 U/L	Exudate if >200 U/L, or pleural-to-serum ratio > 0.6 (Light's criteria)	Exudate
Glucose	45 mg/dL (2.5 mmol/L)	60-100 mg/dL (3.3-5.6 mmol/L); <60 mg/dL indicates a complicated effusion	Reduced
Cholesterol	121 mg/dL (3.1 mmol/L)	Cholesterol-to-triglyceride ratio > 1 suggests pseudochylothorax	Ratio 1.5
Triglycerides	79 mg/dL (0.9 mmol/L)	Chylothorax if >110 mg/dL (1.24 mmol/L)	Below threshold; chylothorax excluded
Bacterial culture	No growth	No growth	Negative
Mycobacterial culture	No growth	No growth	Negative
Fungal culture	No growth	No growth	Negative

**Figure 2:**
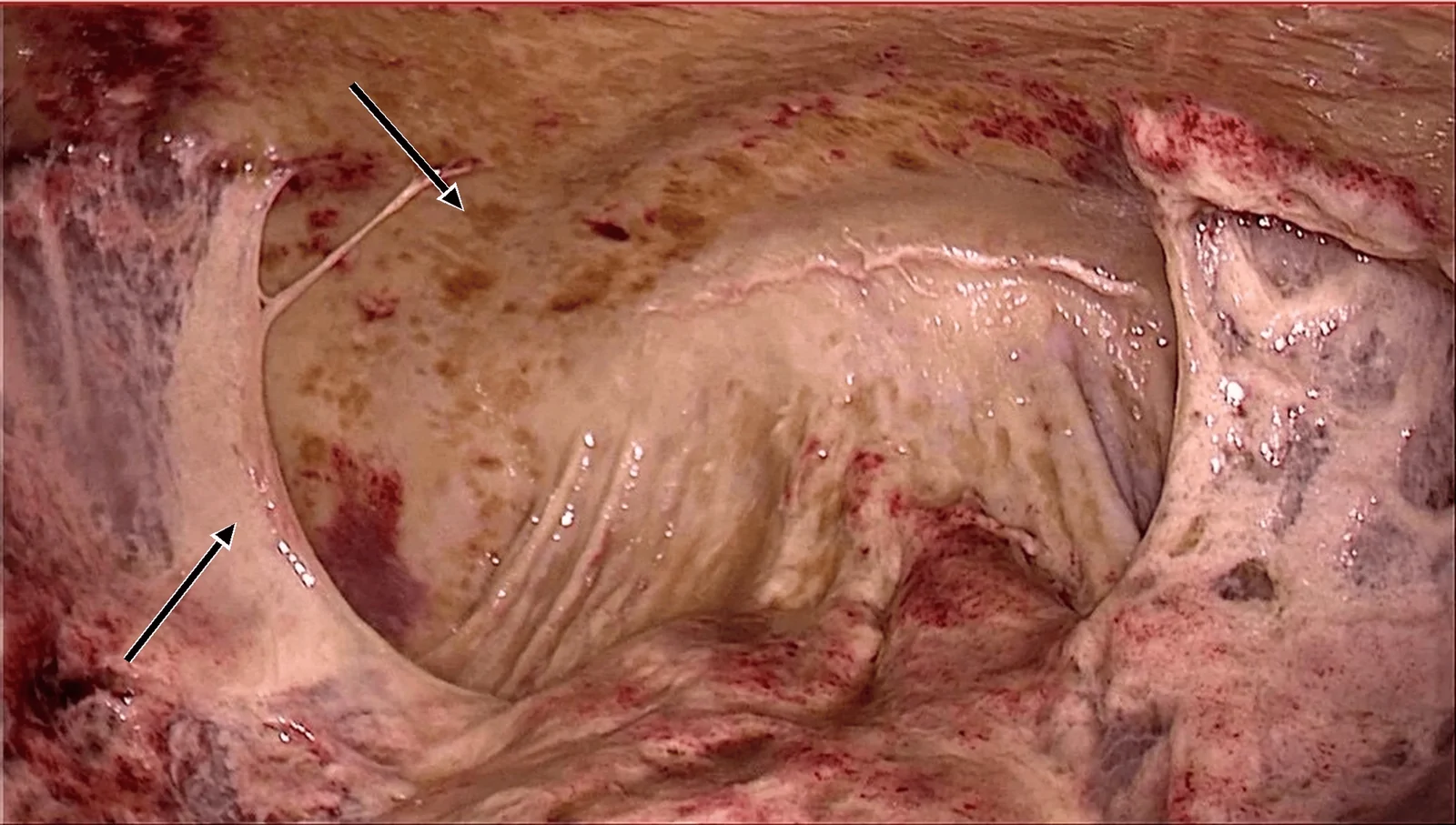
Intraoperative thoracoscopic view of the right hemithorax. The upper arrow indicates diffusely thickened, pale parietal pleura with overlying fibrinous exudate; the lower arrow indicates a band-like fibrous adhesion. Scattered petechial hemorrhage is present

**Figure 3:**
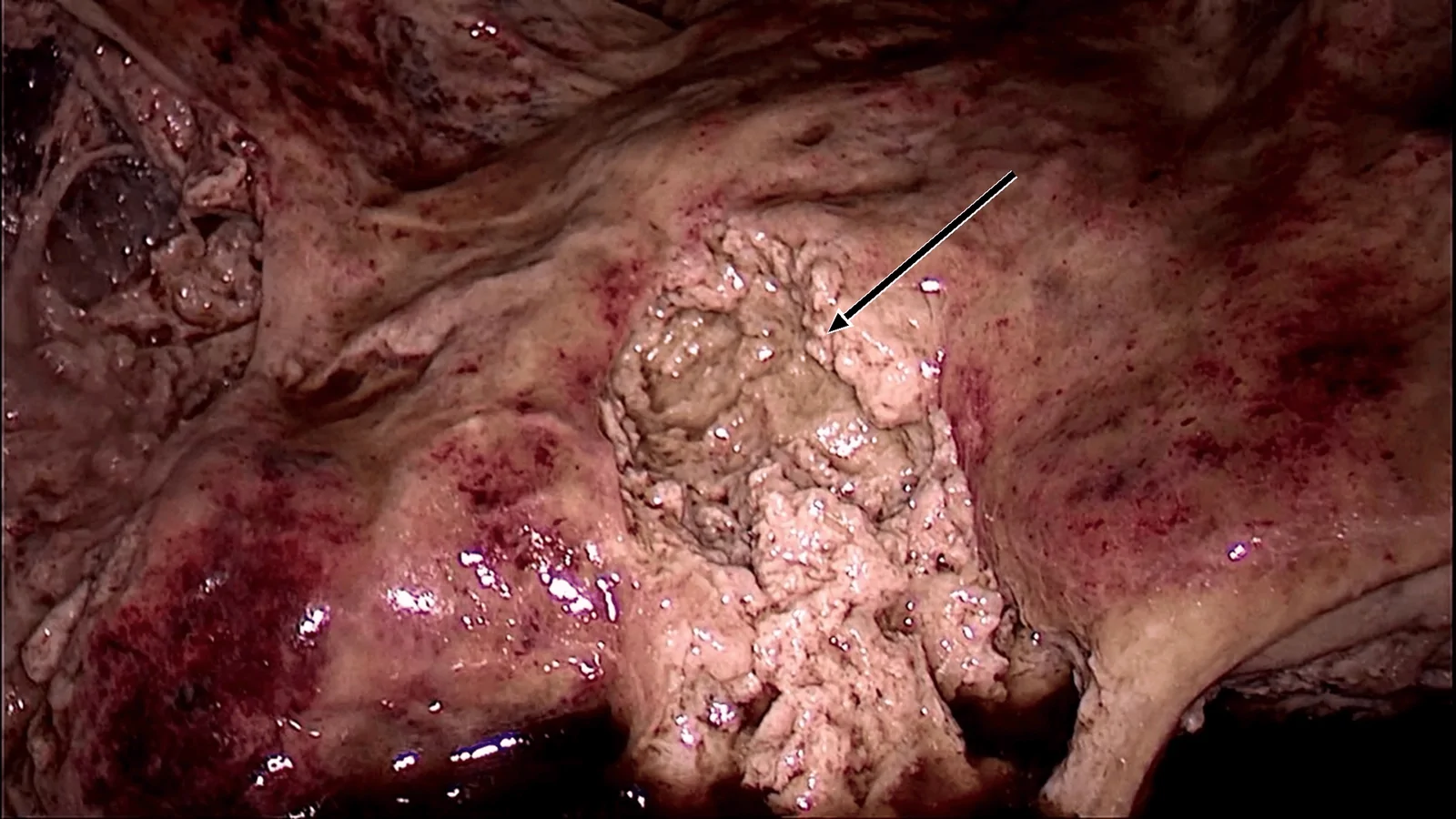
Intraoperative thoracoscopic view showing a raised, irregular, nodular pleural-based lesion consistent with a rheumatoid nodule, surrounded by thickened pleura with petechial hemorrhage

Given the refractory exudative effusion and the absence of a microbiological diagnosis, RA-related pleural disease was suspected. The patient underwent video-assisted thoracoscopic surgery (VATS), which revealed diffuse pleural thickening, fibrinous adhesions, and pleural-based rheumatoid nodules (Figures 2, 3). Histopathological analysis of lung and pleural biopsies confirmed RA-associated fibrinous pleuritis with necrobiotic rheumatoid nodules, follicular bronchiolitis, and a usual interstitial pneumonia (UIP) pattern; there was no caseating granulomatous inflammation, and special stains for organisms were negative. Given the extent of the fibrothorax and trapped lung, staged thoracotomy with decortication and parietal pleurectomy was performed to achieve lung re-expansion and obliterate the pleural space. Concurrently, the immunosuppressive regimen was transitioned from leflunomide to rituximab, in view of concerns regarding leflunomide-associated pulmonary toxicity and the need for an agent with established efficacy in RA-associated interstitial lung disease (RA-ILD).

Detailed follow-up data, including postoperative imaging, pulmonary function testing, and the longer-term response to rituximab, were not available for inclusion in this report.

## Discussion

This case illustrates the diagnostic complexity of RA-associated pleural disease that mimics empyema, particularly in patients with recurrent culture-negative pleural effusions, a recognized but underappreciated clinical pitfall [2,8]. Although most rheumatoid pleural effusions are small and asymptomatic, they may occasionally become large, loculated, and clinically significant [3]. Rheumatoid pleuritis has historically been reported more often in men and in patients with subcutaneous nodules, and it may precede the onset of articular disease [4,9]. The patient's sex was therefore atypical of the classical description; however, more recent reports confirm that rheumatoid pleural effusion also occurs in women and across a broader age range [10].

The biochemical profile of a rheumatoid pleural effusion substantially overlaps with that of empyema. Both may be exudative and may demonstrate a low pH, low glucose concentration, elevated LDH, and early neutrophil predominance [3,8]. In this patient, the turbid green fluid further suggested infection. However, repeated bacterial, mycobacterial, and fungal cultures remained negative. A markedly inflammatory, culture-negative pleural effusion in a patient with established RA should therefore prompt consideration of rheumatoid pleuritis [3,10]. Nevertheless, pleural infection can present with identical biochemical findings, so antimicrobial therapy and drainage remain appropriate until infection has been adequately excluded [8].

Pleural fluid cytology can provide important diagnostic clues. Elongated macrophages, multinucleated giant cells, and granular necrotic debris are characteristic findings and may be highly suggestive of rheumatoid pleuritis [8,9]. The pus-like appearance may result from cellular debris arising from the necrobiotic core of rheumatoid nodule-like granulomas, making the fluid visually indistinguishable from purulent material [5]. In this case, a definitive diagnosis was established only after video-assisted thoracoscopic biopsy demonstrated fibrinous pleuritis, follicular bronchiolitis, and a UIP pattern [11-13]. The combination of sterile inflammatory pleural fluid, pleural-based rheumatoid nodules, and compatible airway and parenchymal histopathology established the diagnosis.

Small airway involvement in RA includes follicular bronchiolitis and constrictive bronchiolitis [1,14,15]. Follicular bronchiolitis is characterized by hyperplastic lymphoid follicles with reactive germinal centers within the bronchiolar walls, reflecting chronic antigenic stimulation [14]. UIP is the most common histopathological pattern of RA-ILD and is associated with an unfavorable prognosis [15,16]. In a population-based cohort, median survival after the diagnosis of interstitial lung disease in patients with RA was 2.6 years, and mortality was approximately threefold higher than in patients with RA without interstitial lung disease [16]. Neither UIP nor follicular bronchiolitis is specific to RA; therefore, interpretation requires integration of the clinical presentation, serologic background, pleural findings, microbiology, and histopathology.

Management differs fundamentally between empyema and rheumatoid pleural disease. Empyema requires antimicrobial therapy and drainage, with VATS or intrapleural enzyme therapy considered for organized pleural infection [11,17]. In contrast, rheumatoid pleural effusions are primarily treated by controlling the underlying autoimmune disease [3,8]. Non-steroidal anti-inflammatory drugs, systemic corticosteroids, and, in selected refractory cases, intrapleural corticosteroids have been reported [3,8]. When chronic loculation produces fibrothorax, restrictive physiology, or a trapped lung, surgical decortication may be required [11]. In this patient, the advanced fibrothorax necessitated thoracotomy, decortication, and parietal pleurectomy. This course highlights the potential consequences of delayed recognition, including progression to fibrothorax and trapped lung requiring extensive surgical intervention.

The transition from leflunomide to rituximab was clinically reasonable for two reasons. First, leflunomide has been associated with drug-induced interstitial lung disease, particularly in patients with pre-existing pulmonary disease [18]. Because this patient had also received methotrexate for 11 years, the relative contributions of RA itself, chronic inflammation, and prior antirheumatic therapy to the pulmonary abnormalities could not be determined with certainty. Second, rituximab has been associated with stabilization or improvement of lung function in RA-ILD and has an acceptable safety profile in this setting [19]. Other biologic agents, including abatacept, have also been used in RA-ILD [15]. More broadly, pulmonary disease in patients with immune dysregulation can present substantial diagnostic and therapeutic challenges. Opportunistic pulmonary infection may occur in patients with underlying immune dysfunction, as illustrated by a reported case of de novo *Pneumocystis jirovecii* pneumonia in a patient with a telomere biology disorder [20]. In addition, treatment failure related to biologic immunogenicity has been described in refractory inflammatory lung disease, including sarcoidosis associated with infliximab-neutralizing antibodies [21].

This report has limitations inherent to a single-patient description. The retrospective nature of the clinical data precludes causal conclusions regarding the effects of surgery or rituximab. Pleural fluid pH, nucleated cell differential, rheumatoid factor, complement levels, and adenosine deaminase were not available for review. Although the combination of sterile inflammatory fluid and characteristic histopathology in a patient with established seropositive RA strongly supported the diagnosis, these additional tests would have strengthened the diagnostic assessment and assisted further in excluding tuberculous pleuritis. Detailed postoperative imaging, pulmonary function testing, and long-term clinical follow-up were also unavailable, limiting assessment of the durability of the response to decortication and rituximab.

## Conclusions

This case highlights the diagnostic challenge posed by RA-associated pleural disease presenting as a recurrent, culture-negative effusion that mimics empyema. In patients with RA and pleural effusion, particularly when cultures are negative and systemic signs of sepsis are absent, an autoimmune etiology should be actively considered alongside continued exclusion of infection. Early recognition, targeted investigation including VATS biopsy where indicated, and timely escalation of immunosuppressive therapy may reduce the risk of complications such as trapped lung and the need for extensive surgical intervention. Multidisciplinary collaboration among rheumatology, pulmonology, and thoracic surgery is essential to optimize patient outcomes.
